# Experimental Study of the Implantation Process for Array Electrodes into Highly Transparent Agarose Gel

**DOI:** 10.3390/ma17102334

**Published:** 2024-05-14

**Authors:** Shengjie Wang, Xuan Yan, Xuefeng Jiao, Heng Yang

**Affiliations:** Beijing Key Laboratory of Lightweight Multi-Functional Composite Materials and Structures, Institute of Advanced Structure Technology, Beijing Institute of Technology, Beijing 100081, China; 3120211961@bit.edu.cn (S.W.); jiaoxf1@163.com (X.J.)

**Keywords:** array electrode, implantation process, digital gradient sensing, stress state

## Abstract

Brain–computer interface (BCI) technology is currently a cutting-edge exploratory problem in the field of human–computer interaction. However, in experiments involving the implantation of electrodes into brain tissue, particularly high-speed or array implants, existing technologies find it challenging to observe the damage in real time. Considering the difficulties in obtaining biological brain tissue and the challenges associated with real-time observation of damage during the implantation process, we have prepared a transparent agarose gel that closely mimics the mechanical properties of biological brain tissue for use in electrode implantation experiments. Subsequently, we developed an experimental setup for synchronized observation of the electrode implantation process, utilizing the Digital Gradient Sensing (DGS) method. In the single electrode implantation experiments, with the increase in implantation speed, the implantation load increases progressively, and the tissue damage region around the electrode tip gradually diminishes. In the array electrode implantation experiments, compared to a single electrode, the degree of tissue indentation is more severe due to the coupling effect between adjacent electrodes. As the array spacing increases, the coupling effect gradually diminishes. The experimental results indicate that appropriately increasing the velocity and array spacing of the electrodes can enhance the likelihood of successful implantation. The research findings of this article provide valuable guidance for the damage assessment and selection of implantation parameters during the process of electrode implantation into real brain tissue.

## 1. Introduction

Brain–computer interface (BCI) technology allows direct communication between the brain and external devices; it has found applications in various fields, including medical [1,2,3], entertainment [4], and military [5]. Research in BCI technology not only deepens our understanding of brain mechanisms but also develops new approaches for treating brain disorders. Implantable electrodes are a key component of BCI systems as they directly interface with the neural tissue of the brain to capture the electrical signals generated by brain activity [6,7,8]. However, the implantation of electrodes in the brain can cause severe inflammation and damage to brain tissue due to immune or nonimmune reactions, such as tissue damage caused by surgical procedures [9,10,11,12]. Among them, strain and stress around the implantation site during electrode implantation are the main factors causing sustained brain tissue reactions, potentially exacerbating brain inflammation. Therefore, studying the process of electrode implantation into brain tissue is of positive significance for improving brain tissue injury and increasing the likelihood of successful implantation [13].

Predicting the load during electrode implantation into brain tissue is crucial for improving the safety, effectiveness, and reliability of implantation procedures [14,15,16,17]. By conducting implantation puncture mechanics experiments, we can assess the conditions necessary for successful electrode implantation, predict potential failure risks, and gain insights into the forces exerted on brain tissue during electrode implantation. This understanding is vital as it informs the development of strategies to enhance surgical outcomes. In order to investigate the load on brain tissue during electrode implantation, Wittek et al. [18] used solid stainless-steel needles with a diameter of 1.15 mm to implant into a porcine brain, focusing on the insertion phase before brain dura mater puncture, and explored the trend of insertion forces on the tissue during needle implantation. Jiang et al. [19] conducted a series of needle-puncture experiments on soft tissues to explore the effects of electrode size, insertion speed, driving mode, and insertion type on the implantation load, which helps to determine the electrode position and provide an effective implantation strategy.

Building on these foundational studies, predicting the damage that may occur during electrode implantation into brain tissue becomes an essential step to take appropriate measures to reduce surgical risks and effectively minimize postoperative discomfort and complications for patients [20,21,22,23,24]. Urrea et al. [25] examined the implantation process of stainless-steel hollow needles with different outer diameters and speeds, predicting potential damage by evaluating the friction coefficient between the needles and hydrogel. Zhang et al. [26] developed an evaluation system based on a microscopy digital image correlation method for detecting brain tissue damage caused by neural probe insertion. The system can extract tissue deformation information from the captured speckle patterns, assessing the brain tissue damage induced by the neural probe. Despite certain advancements in related research, real-time observation of brain tissue damage during high-speed or array implantation processes remains a significant challenge. A particularly notable gap in the current research is an in-depth analysis of how the spacing of electrode arrays impacts the implantation process. Such analysis is crucial for optimizing implantation techniques, reducing the risk of brain tissue damage, and enhancing the success rate of implant surgeries. Therefore, this study systematically evaluates the specific effects of electrode array spacing on the implantation process through meticulous experimental design and advanced imaging technology.

In this study, we have constructed a digital gradient sensing (DGS) experimental setup capable of in situ observation to reveal the load and damage during the array electrode implantation process into the highly transparent agarose gel. Firstly, we prepared the transparent agarose gel, which facilitated real-time observation throughout the entire implantation process. Then, the hyper-viscoelastic properties of the agarose gel were characterized through compression and indentation experiments. The results confirmed that this material exhibited mechanical properties analogous to those of authentic brain tissue. Finally, experiments were conducted on the agarose gel, in which both single and array electrodes were implanted under various conditions to reveal the potential damage.

## 2. Experimental Description

### 2.1. Materials

The low-melting-point agarose powder (T1050), 5X TBE buffer (2276GR025), Deionized water, and heating magnetic stirrer (HJ-6) are purchased from Solarbio, BioFroxx, Xizhimeng Trade Co., Ltd. (Shuyang, China) and Jiangsu KeXi Instrument Co., Ltd. (Changzhou, China), respectively. Both the high-temperature-resistant photoresist (HTL) and 3D printer (S140) are provided by Shenzhen Rubik’s Material Technology Co., Ltd. (Shenzhen, China).

### 2.2. Preparation of Highly Transparent Agarose Gel and Electrodes

**Figure 1:**
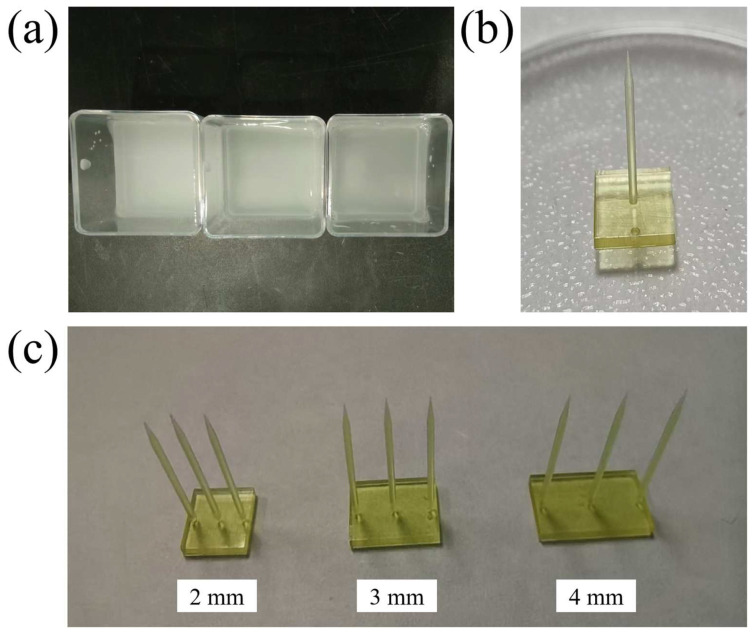
(**a**) Physical image of the transparent agarose gel at 2% concentration, (**b**) the 3D printed single resin electrode, and (**c**) the array resin electrodes under spacing of 2, 3, and 4 mm.

First, combine 5 mL of 5X TBE buffer with 20 mL of deionized water to prepare a total of 25 mL of 1X TBE buffer. Place the prepared 1X TBE buffer in a magnetic stirrer and heat it to 100 °C. Once the temperature is reached, slowly add 0.5 g of low-melting-point agarose powder to the buffer while maintaining a moderate stirring speed with the magnetic stirrer to prevent the agarose from clumping. Record the initial mass of the beaker and the solution. Once the agarose is fully dissolved, measure the amount of water lost to evaporation and replenish it with deionized water, bringing the solution’s total mass back to the original recorded mass and ensuring thorough mixing. Pour the solution into a mold when it cools to 30 °C and allow it to solidify. The resulting 2% transparent agarose gel is shown in Figure 1a.

The electrodes have a length of 10 mm, a diameter of 0.5 mm, and a needle tip angle of 14.25°. Due to the technical challenges and high costs associated with fabricating 10 mm silicon electrodes, we opted for alternative 3D-printed resin electrodes for the implantation experiments. The photopolymer was placed in the resin vat of the 3D printer, and the three-dimensional model of the electrode was imported into the slicing software for processing. The resin electrodes were then fabricated using the 3D printer. To minimize the impact of variances such as surface roughness on our experimental results, we have strictly controlled the manufacturing process. All electrodes were crafted from the same batch, ensuring that the methods and materials used were uniform throughout. As shown in Figure 1b,c, the printed electrodes exhibit good print quality, with needle tip diameters that meet the required specifications.

### 2.3. Uniaxial Compression and Indentation Experiments

**Figure 2:**
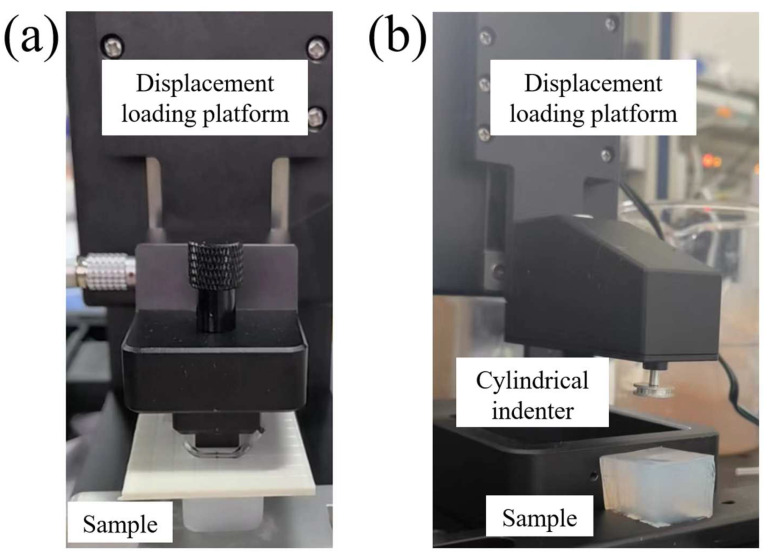
(**a**) Uniaxial compression test procedure; (**b**) indentation test procedure.

The electrode loading testing system (PR-BDM8-100F, Shenzhen Puri Materials Technologies, Co., Ltd., Shenzhen, China) was used for the uniaxial compression and indentation experiments, as shown in Figure 2. For the compression experiments, a one-dimensional force sensor with a range of 3N and an accuracy of 0.03% was utilized. Firstly, the agarose gel was demolded and shaped into a 23 × 20 × 15 mm^3^ cuboid, then placed in the middle position of the loading table plate. The compression was performed at a velocity of 3 mm/min for a displacement of 4.5 mm. After a 400 s holding period, the loading plate returned to its original position at the same velocity.

For the indentation experiments, the loading plate was replaced with a cylindrical indenter with a diameter of 12 mm. The indenter was driven into the agarose gel at a velocity of 2 mm/s, reaching a depth of 1 mm, and then maintained at this depth for a 70 s holding period without any additional displacement. Each experimental group was repeated three times to ensure the reliability of the results.

### 2.4. Electrode Implantation Experimental Setup and DGS Method

DGS is a non-contact measurement technique that integrates digital image correlation (DIC) with the photoelastic effect. The advantage of this method lies in its ability to provide data with high temporal and spatial resolution, which is essential to capture and analyze the deformation and stress variations induced by stress waves propagating through the material [27]. In the electrode implantation experiments, DGS allows us to observe the response of the agarose gel in a full-field, non-contact, real-time manner, which is of significant importance for optimizing the implantation strategy and understanding the mechanical behavior of the material. Benefiting from the high transparency of the agarose gel, DGS can clearly identify scattering patterns at a thickness of 3 mm. The patterns remain unaffected by the incident light, facilitating the observation of stress concentration phenomena. It is worth noting that the DGS system can only be applied to transparent materials, which is one of the main reasons why we chose agarose gel. For opaque real brain tissue, it is difficult for current research methods to observe the damage in real time during the implantation process. The current practice is usually to slice the brain tissue after the implantation experiment to observe deformation and damage. However, since the 2% agarose gel has mechanical properties similar to those of real brain tissue, our study on implantation speed and electrode array spacing still has some guiding significance for clinical applications. The deformation and damage observed in real time during the process of electrode implantation into agarose gel can help optimize surgical techniques, such as the depth and speed of electrode implantation, as well as the selection of electrode size and array spacing.

**Figure 3:**
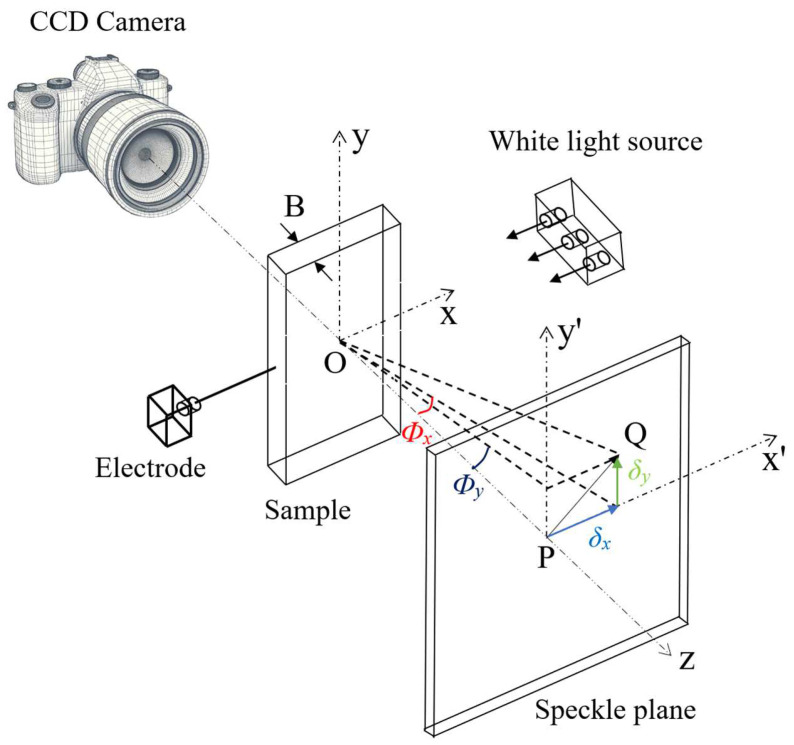
Schematic diagram of the digital gradient sensing (DGS) method’s principle.

Figure 3 illustrates the principle schematic of the DGS method. The camera, transparent sample, and speckle plane are aligned along the same straight line, with the camera focused on the speckle plane. The nominal thickness of the sample is B. A white light source is used to uniformly light up the sample. The in-plane coordinates of the sample and the speckle plane are denoted as (x,y) and (x’,y’), respectively, and their *z*-axis is aligned. When no electrode is implanted, point O on the sample plane corresponds to point P on the speckle plane. Upon electrode implantation, mechanical loading causes changes in the sample’s refractive index and thickness depending on the local stress state, leading to light deflection. That is, after the sample deforms, point O on the sample plane corresponds to point Q on the speckle plane. Assuming the distance between the sample and the speckle plane is Δ, the angle field of the light ray OP relative to the deformed light ray OQ when deformed is:

(1) $$\Phi_{x}\approx \frac{\delta_{x}}{△}\approx C_{\sigma }B\frac{\partial \left(\sigma_{xx}+\sigma_{yy}\right)}{\partial x}$$

(2) $$\Phi_{y}\approx \frac{\delta_{y}}{△}\approx C_{\sigma }B\frac{\partial \left(\sigma_{xx}+\sigma_{yy}\right)}{\partial y}$$

(3) $$C_{\sigma }=D_{1}-\upsilon (n-1)/E$$

Here, $C_{\sigma }$ denotes the elastic optical coefficient of transparent material, $D_{1}$, υ, n, and E represent the photoelastic coefficient, poisson’s ratio, refractive index, and elasticity modulus, respectively. The terms $\partial \left(\sigma_{xx}+\sigma_{yy}\right)/\partial x\;$and $\partial \left(\sigma_{xx}+\sigma_{yy}\right)/\partial y$ correspond to the spatial gradients of two orthogonal stress components $\left(\sigma_{xx}+\sigma_{yy}\right)$, along the x and y directions, respectively. Additionally, $\sigma_{xx}$ and $\sigma_{yy}$ are defined as the stresses along the x and y directions, respectively.

**Figure 4:**
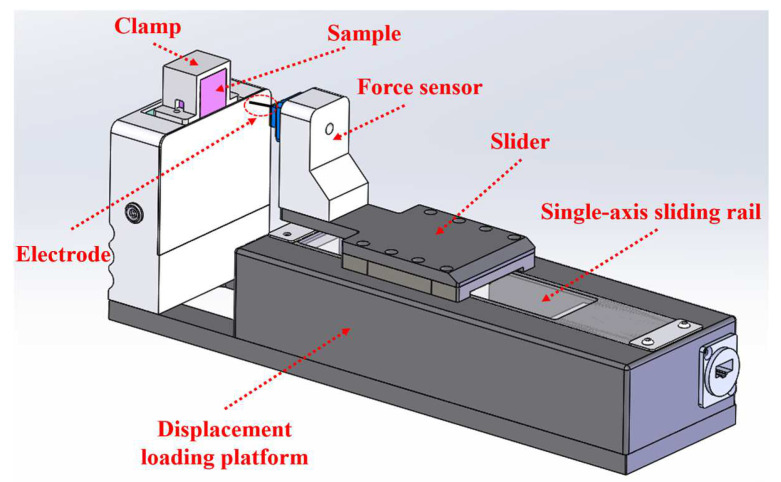
Schematic diagram of the displacement loading platform and implantation method.

Figure 4 depicts a schematic diagram of the displacement loading platform and the implantation method. The sample is held in place by a clamp made of transparent acrylic material, allowing for unobstructed observation. The electrode is strategically placed at the center of the force sensor’s end to ensure precise alignment during implantation. This force sensor is securely attached to a slider, enabling it to move smoothly along a single-axis sliding rail at a uniform speed. This configuration is crucial for achieving consistent and accurate implantation procedures.

**Figure 5:**
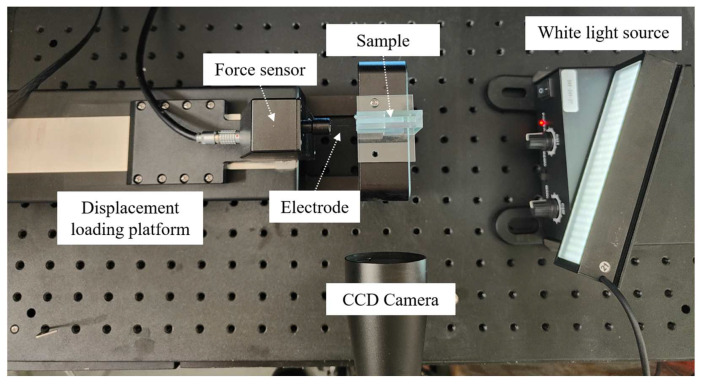
Electrode implantation into the highly transparent agarose gel experimental setup.

Figure 5 illustrates the electrode implantation into the highly transparent agarose gel experimental setup. Use the aforementioned electrode loading testing system to apply a constant velocity load to the electrode. A 25 W white light source was positioned at a sufficient distance from the sample to minimize the effects of heat flow. This helps to prevent a reduction in the contrast and clarity of the speckle images, thereby enhancing the measurement precision of the experimental setup. The charge-coupled device (CCD) camera (MV-CE200-11UM, Hikrobot Technology Co., Ltd., Hangzhou, China) parameters were carefully chosen to provide a field of view measuring 18 mm by 12 mm, ensuring focus on the speckle plane. The photo, with a resolution of 5472 pixels by 3648 pixels, results in an individual pixel size of 3.3 μm. The resolution of the DIC method can realize the precision with 0.01 pixel [28]; therefore, the in-plane displacement resolution is approximately 33 nm. Additionally, make sure that the key features of the sample, such as edges, are clearly visible in the captured image. This will aid in conducting accurate analysis during the subsequent image processing phase. The speckle plane was created by printing after generating random speckle patterns with software that specified a density of 50%, a diameter of 0.05 mm, and a randomness level of 50%.

### 2.5. Electrode Implantation Experimental Protocol

The transparent agarose gel was precisely cut into a cuboid, measuring 3 × 30 × 40 mm^3^. To secure the agarose gel and electrodes at the specific locations required by the electrode loading setup of the force testing system, a specially designed fixture with a thickness of 3 mm was employed. The setup ensured a distance of 13 mm between the camera and the speckle patterns, with the specimen placed 4 mm away from the speckle patterns. For the speckle pattern noted, the resolution of in-plane displacement that can be detected is 3.3 μm. Due to the high transparency of the agarose gel, which allows light to easily penetrate through it, this enables the camera to accurately adjust the focus, resulting in a clear image of the speckle pattern plane on the image sensor. During the experiment, the displacement loading platform moved at a consistent velocity, allowing the CCD camera to capture the electrode implantation process at a rate of 20 frames per second. Additionally, we turned off the auto-exposure mode and manually set the exposure time to 0.005 s. To minimize experimental variability, each condition within the experimental setup was replicated three times. The implantation of single electrodes was performed at varying velocities of 0.2, 1, 2.63, and 5 mm/s. For array electrode implantation experiments, where the inter-electrode spacing was set at 2, 3, and 4 mm, a uniform velocity of 2.63 mm/s was maintained.

## 3. Results and Discussion

### 3.1. Mechanical Properties of Agarose Gel

**Figure 6:**
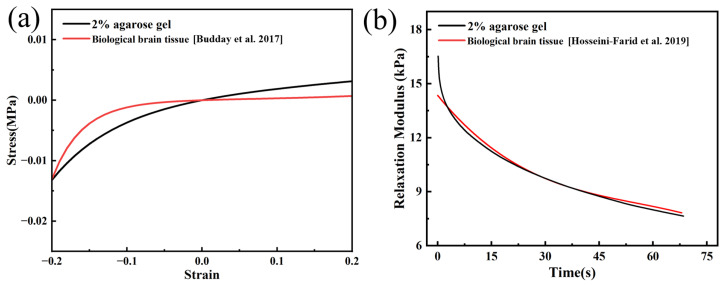
(**a**) Stress-strain [29] and (**b**) relaxation modulus-time curves of 2% agarose gel and actual brain tissue [30].

Acquiring biological brain tissue is a task fraught with difficulties and ethical considerations, posing significant challenges to research. Even if biological brain tissue can be successfully obtained, the sample timeliness and variability in in vitro experiments may lead to unstable results, thereby increasing the complexity of the research. When conducting implantation experiments with biological brain tissue, it is not possible to observe potential tissue damage in real time. Typically, the extent of implant damage can only be assessed by examining brain tissue sections post-experiment. However, this approach may fail to capture specific instances or acute injuries. Therefore, our study chose agarose gel as an alternative to biological brain tissue for electrode implantation experiments. By adjusting the ratios, the gel could simulate mechanical properties similar to those of brain tissue. In addition, the high transparency of agarose gel enabled real-time observation of damage and deformation during implantation. To verify the mechanical properties of the agarose gel, uniaxial compression, and indentation, experiments are conducted, with each designed to characterize its hyperelastic and viscoelastic behaviors, respectively. As shown in Figure 6 for the comparison between the experimental fitting results of this study and the experimental results of actual brain tissues in the literature [29,30], it can be observed that the mechanical properties of the 2% agarose gel prepared in this study are similar to those of actual brain tissues. Within the range of 0–0.2 compressive strain, the compressive modulus brain tissue varies from 10.1 to 266.7 kPa, while the compressive modulus of our prepared 2% agarose gel ranges from 30.3 to 142.9 kPa. It can be observed that the range of the compressive modulus for the 2% agarose gel significantly overlaps with that of the actual brain tissue, indicating that our prepared gel can simulate the mechanical properties of brain tissue with considerable accuracy. Although the specific values are not entirely the same, the similarity in the range of the compressive modulus highlights the advantage of using 2% agarose gel as a model material for studying the mechanical behavior of brain tissue. Although agarose gels cannot fully mimic all the characteristics of brain tissue, some similar mechanical behaviors between them provide our study with some reference value for the selection of electrodes and experimental design during the implantation process into real brain tissue.

### 3.2. Single Electrode Implantation Experiments

Before determining the boundary conditions for experimental testing, we conducted a thorough finite element simulation analysis to assess the impact of boundary conditions such as the clamping method and the size of the agarose on the experimental outcomes. Our established finite element simulation model took into account the mechanical behavior of the probe and the agarose, under the same boundary conditions as the experimental tests. Based on the finite element simulation model, we calculated the deformation evolution under various implantation speeds. By comparing with the strain cloud map of the agarose shown in Figure S1, it was observed that the material deformation near the boundaries of the agarose was minimal across different implantation speeds. This suggests that the selection of agarose size and the clamping method during the experimental testing process does not influence the deformation evolution of the brain tissue during implantation or the interpretation of the test results. Furthermore, the consistency between the experimental and simulation results further validates the rationality and reliability of our simulations, as depicted in Figure S2.

It is worth noting that for electrode implantation experiments, the differences between resin electrodes and the silicon or metal electrodes commonly used in clinical practice are mainly reflected in their different moduli. However, the modulus of each type of electrode is much greater than that of agarose gel, and they can be approximated as rigid bodies. In clinical applications, to prevent signal interference between different electrodes, a layer of insulating material, such as Parylene, is commonly added to the surface of silicon or metal electrodes. Moreover, our study primarily focuses on the impact of electrode implantation on the macroscopic mechanical properties of agarose gel, where the conductivity of the electrode has a minimal effect on the tissue damage during the implantation process. Therefore, despite the differences in material properties between resin electrodes and silicon or metal electrodes, the resin electrode is still representative in studying the mechanical behavior of agarose gel during the implantation process.

**Figure 7:**
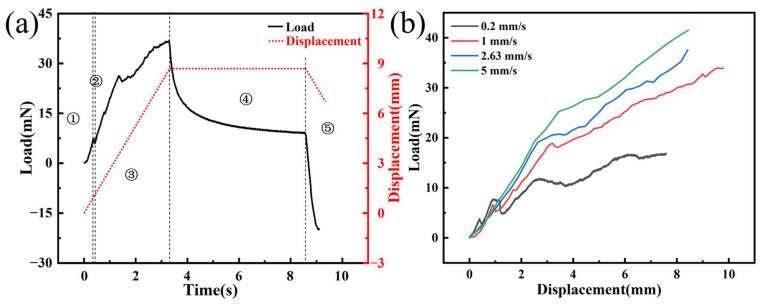
(**a**) Load–time curve of single electrode implantation experiment at 2.63 mm/s (①: begin implantation; ②: puncture moment; ③: continuous puncture; ④: implantation stop; ⑤: electrode extraction). (**b**) Load–displacement curves of single electrode implantation experiment at different speeds.

Figure 7a shows the time–load curve of a single electrode implanted into agarose gel at a speed of 2.63 mm/s, which reveals five distinct phases. Firstly, the initial phase is marked by the deformation of the agarose gel from the moment the needle tip makes contact until right before the puncture occurs. The load applied to the electrode in this phase originates from the elastic deformation of the agarose gel, and the electrode load steadily increases due to the gradual increase in the strain energy of it. Then, the punctured stage is entered, which is typically characterized by a sharp drop in the electrode load due to the release of strain energy from the agarose gel when it is punctured. The curve clearly shows a distinct peak, which is consistent with the findings of Welkenhuysen et al. [20], indicating that the peak is caused by tissue penetration by the electrode. Meanwhile, we recorded the entire process of electrode implantation using a CCD camera. By comparing the curve with the images captured during the experiment, we confirmed that the peak phenomenon is indeed caused by the electrode puncturing the agarose gel. The third phase is the steady-state puncture stage when the load mainly comes from the friction between the needle axis and the agarose gel, the stiffness force of the tissue, and the puncture force. As the needle tip continues to puncture, the contact area between the needle axis and the agarose gel increases, resulting in a steady increase in load. Subsequently, the tissue relaxation phase begins when the electrode is implanted at the designated position and stops. Due to the viscoelasticity of the agarose gel, the release of strain energy leads to a rapid decrease in electrode load. Finally, in the electrode extraction phase, the electrode returns at a constant speed of 2.63 mm/s, and the load is mainly composed of friction.

Figure 7b illustrates the load–displacement curves at different insertion speeds, revealing that with increasing velocity, the implant load also increases correspondingly. When the implantation depth is 7 mm, the implantation forces of the electrode are 16.46, 27.82, 31.12, and 36.13 mN, with implantation velocities of 0.2, 1, 2.63, and 5 mm/s, respectively. This trend can be attributed to the rate-dependent stiffening behavior of the material, where the material’s resistance augments as the insertion velocity is enhanced. At higher application speeds, agarose gel predominantly exhibits its elastic behavior, leading to an increased resistance to deformation and, consequently, a more pronounced rigidity characteristic. Specifically, as the implantation speed increases, the viscoelastic response of agarose gel relatively weakens. This is primarily because, under rapid application of force, the gel’s viscous component struggles to absorb the applied force through internal flow or energy dissipation, making the elastic component the dominant response mechanism. This shift in mechanism enhances the gel’s resistance to penetration attempts, reflecting its adjustment to mechanical properties under high-speed implantation conditions. However, we observed that when the implantation speed was 0.2 mm/s, the load–displacement curve was relatively flat within the range of approximately 2.5–5 mm. Due to the viscoelasticity and stick-slip effect of the agarose gel, there is a sudden drop in load at the moment the electrode punctures the agarose gel. As the electrode continues to be implanted, the load gradually increases. Thus, the initial decrease followed by a rise in load depicted in the graph is caused by the continuous puncture of the agarose gel by the electrode tip. The phenomenon is more pronounced when the implantation speed is low, but it diminishes as the implantation speed increases.

**Figure 8:**
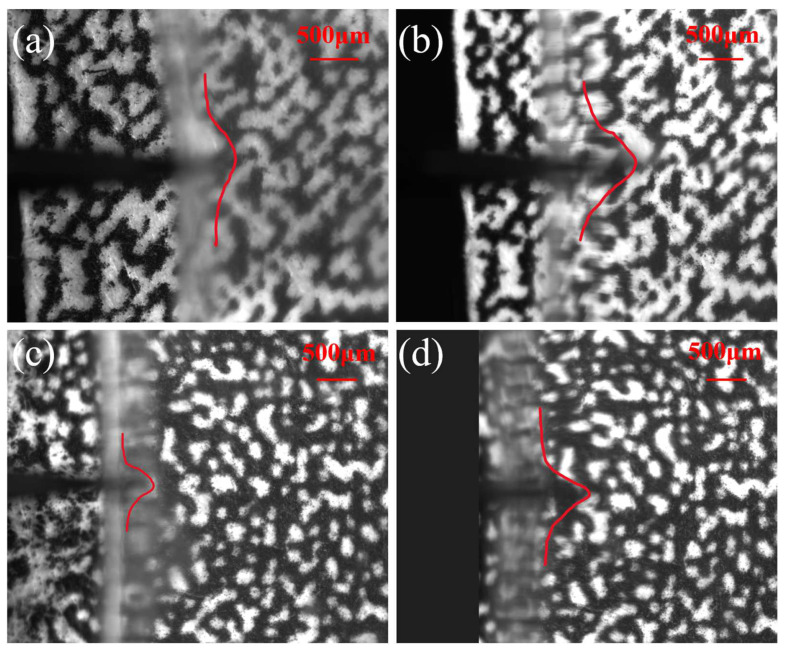
The moment the agarose gel was punctured when the implantation speeds are (**a**) 0.2, (**b**) 1, (**c**) 2.63, and (**d**) 5 mm/s, respectively and the red line represents the deformation profile of the agarose gel.

The puncture angle refers to the angle formed at the tissue surface the instant the electrode pierces through it. To minimize tissue damage and reduce bleeding, inflammation, and cell death caused by the implantation process [31], the ideal scenario is to pursue the smallest possible puncture angle. Figure 8 illustrates the moment when the agarose gel is penetrated at different implantation speeds, clearly demonstrating a trend: as the implantation speed increases, the puncture angle gradually decreases. The puncture angles are 129.54°, 97.43°, 84.36°, and 66.89°, at implantation speeds of 0.2, 1, 2.63, and 5 mm/s, respectively. This is due to the viscoelastic properties of the material; at lower implantation speeds, strain typically affects a wider area. However, as the implantation speed increases, the range of strain gradually narrows, resulting in more localized damage. It is worth noting that in brain–computer interface research, the issue of electrode implantation is an important research direction that spans multiple disciplines. The “damage” defined in our study primarily considers macroscopic injury to brain tissue, which is different from the microscopic injuries discussed in the field of neurology, such as blood vessel rupture, bleeding, neuronal cell death, and tissue necrosis. Therefore, based on our research focus, which is on the mechanical macroscopic injury issues during the implantation process, we have chosen agarose gel with mechanical properties similar to real brain tissue to simulate the macroscopic injury issues of real brain tissue during the electrode implantation process. The internal biological microscopic injuries caused by macroscopic damage to brain tissue during the implantation process require further analysis and characterization through multidisciplinary research methods.

**Figure 9:**
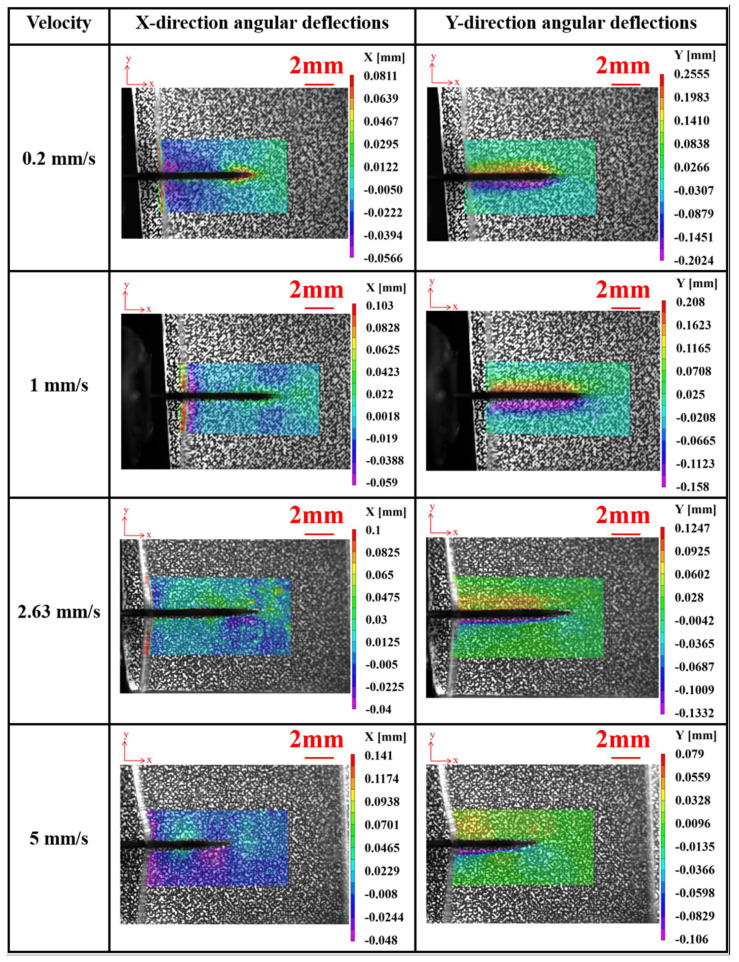
The X and Y direction angular deflections of agarose gel in single electrode implantation experiments at various speeds.

Through the utilization of the digital image correlation method, we conducted a quantitative analysis of the implantation process, which not only corroborated the visual observations but also facilitated a detailed examination of the stress distribution. Figure 9 illustrates the results of angular deflection measurements of the agarose gel in the X and Y directions during implantation with a single electrode at various speeds. The data reveal significant angular deflection near the needle tip and along its axis, indicating stress concentration phenomena. Conversely, minimal angular deflections are observed at the plot boundaries, suggesting the absence of boundary effects. This finding underscores the reliability of our experimental setup.

To provide a comprehensive understanding, Supplementary Video S1 records the complete implantation process at an insertion speed of 2.63 mm/s. The video vividly showcases the deformation process, offering valuable insights into the experimental procedure. Combined with the observable depression of the electrode during steady-state implantation in the 3 mm thick agarose gel, the validation of our experimental setup suggests that the selected dimensions are suitable for this study. This observation provides valuable insights for future research in this field.

**Figure 10:**
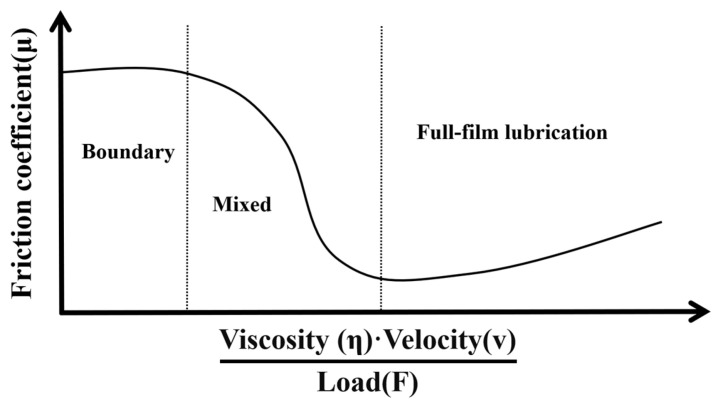
Schematic of a Stribeck curve.

Furthermore, under the same implant displacement, the degree of depression in the agarose gel was observed to increase progressively with the enhancement of implantation velocity. The angular deflection, as indicated by the graph, exhibits a radial distribution, with the radiating phenomenon becoming more distinct as the implantation speed increases. This can be attributed to the increased kinetic energy at the needle tip with rising velocities, which amplifies its impact on the agarose gel. However, the regions of damage within the agarose gel appear to decrease as the implantation speed escalates which is a common reason for preferring higher speeds in electrode implantation practices [32]. Due to rapid loading, the stiffness of the agarose gel increases, making it more resistant to deformation. This phenomenon can be explained from two aspects. Firstly, the higher the speed, the smaller the puncture angle becomes, resulting in more localized damage to the agarose gel. Secondly, according to the theory of the Stribeck curve in the field of tribology, due to the high water content of the agarose gel created for this study, the friction between the electrode and the agarose gel exists in a state between boundary and mixed lubrication, as shown in Figure 10. In such a state, the greater the speed, the lower the coefficient of friction between the surfaces in contact.

When implanting electrodes at high speed, the impact on the tissue is instantaneous and concentrated, allowing the electrodes to quickly penetrate the agarose gel, reducing the movement of the surrounding tissue, and thus localizing the damage. However, this rapid penetration also results in a greater mechanical pressure being exerted on the local area, which may increase the severity of the damage. Therefore, although the area of damage is more focused, the extent of the damage may intensify due to the increased mechanical force. Therefore, when planning and conducting implantation experiments, it is crucial to comprehensively consider the dual effects of implantation speed on load increase and potential damage to find an optimal implantation speed that balances minimizing damage and ensuring effective implantation. In our experimental results, we observed that when the implantation speed was 2.63 mm/s, the maximum angular deflections of the sample in both the x and y directions were the smallest relative to other implantation speeds, indicating the least degree of damage. Additionally, the area of damage was predominantly localized around the needle tip and along the needle shaft, as depicted in Figure 9. When the implantation speed was increased to 5 mm/s, it was evident that both the extent of damage and the maximum angular deflections had increased. Therefore, we consider 2.63 mm/s to be the appropriate implantation speed. This finding provides valuable reference for future research by other scholars in this field.

### 3.3. Array Electrode Implantation Experiments

A single electrode is capable of measuring only limited signals in the vicinity of its tip. Even with multiple recording points on the electrode, these points can only capture brain electrical activity along the electrode’s axis. This constrains the ability to thoroughly record brain electrical signals on the same horizontal level. Hence, to achieve a more comprehensive gathering of brain electrical signals for the purposes of signal decoding and analysis, it is common practice to implant several electrodes simultaneously during animal or human experiments.

**Figure 11:**
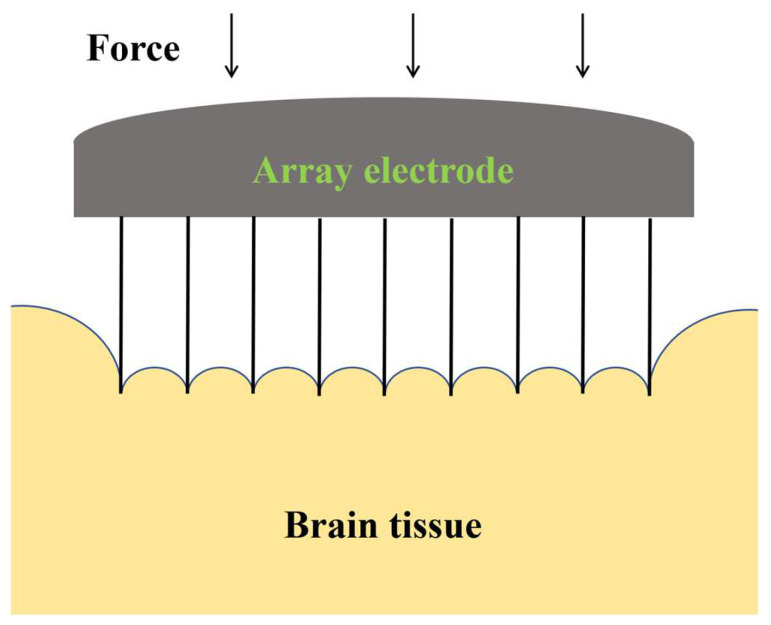
The coupling effect of array electrode implantation.

Unlike single electrodes, the spacing between electrodes in an array is a critical factor that affects both the health of brain tissue and the quality of signal acquisition. If the electrodes are too closely spaced, the coupling effect between adjacent electrodes may cause excessive damage to the brain tissue, as shown in Figure 11. Conversely, if the spacing between electrodes is too wide, it may not capture all necessary signals within the region. Therefore, studying the optimal spacing of electrode arrays is crucial for achieving precise signal collection and minimizing brain tissue damage. Considering the Utah electrode’s well-established use in clinical settings, where the inter-electrode spacing is conventionally set at five times the diameter of the individual electrodes, such as those sold by Blackrock, we tailored our experimental design accordingly. Given that the diameter of the electrodes we are using in our study is 0.5 mm, we have established the distances between electrodes for our implantation experiments as 2, 3, and 4 mm. This decision was made to align with the clinical standards while accommodating the specific requirements of our research.

**Figure 12:**
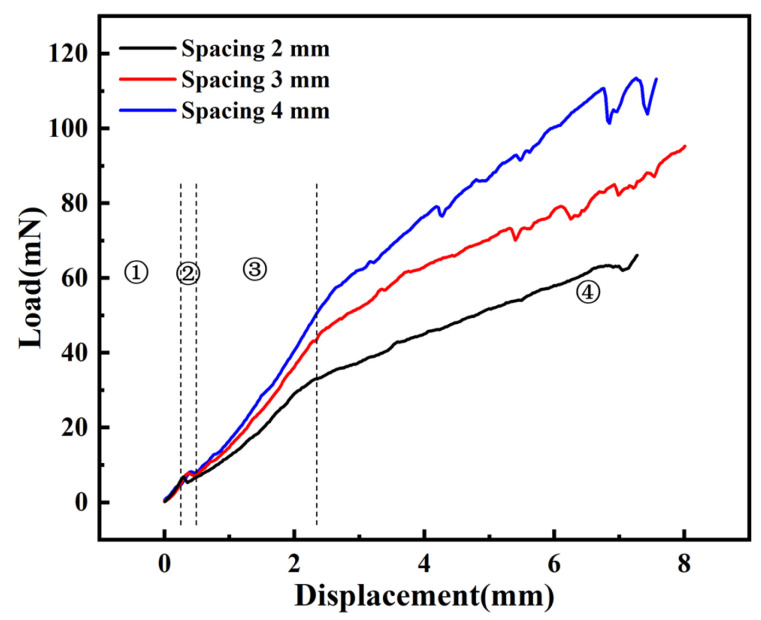
Load–displacement curves for different spacing array electrode implantation experiments at a speed of 2.63 mm/s (①: begin implantation; ②: puncture moment; ③: electrode tip implantation; ④: electrode shaft implantation).

Figure 12 presents the load–displacement curves for array electrode implantation experiments at an insertion speed of 2.63 mm/s, with varying electrode spacings, from the initial contact of the electrode array with the agarose gel to the cessation of implantation. The load–displacement curves can be divided into four distinct phases. The first phase is akin to the single electrode implantation experiment, with the load continuously increasing from the moment the electrode first comes into contact with the agarose gel until just before the puncture. During the second phase, a noticeable reduction in the peak amplitude of the load–displacement curve is observed compared to that of a single electrode, which may be attributed to the distribution and interaction of the array electrodes. By analyzing images captured during the implantation process by a CCD camera, we observed that the vibration of the array electrodes during implantation is reduced compared to a single electrode. Supplementary Video S2 shows the complete implantation process for an array spacing of 4 mm. The third phase is identified where the electrode tips penetrate the agarose gel, starting from the initial puncture of the gel by the tips and continuing until all of the conical tips are fully embedded within the gel. The fourth phase represents the steady-state implantation process, where the diameter of the electrode remains constant. In this stage, as the electrode continues to be implanted, the agarose gel further depresses, and the load gradually increases, until the completion of implantation. The experimental results indicate that an increase in electrode spacing leads to a rise in implantation load at the same implantation depth. When the implantation depth is 8 mm, the implantation load increases from 61 mN to 115 mN, an increase of 88.5% when the electrode spacing is increased from 2 mm to 4 mm.

According to the research by Davidson et al. [33], experiments with hollow microneedle arrays for transdermal drug delivery showed that increasing the spacing between arrays leads to an increase in the surface area of each microcirculation interface and lengthens the diffusion path from the center of the microneedle to the interface corners. Consequently, at the same depth of implantation, as spacing increases, the contact area between the agarose gel and the electrode array grows, resulting in a higher implantation load and making it more challenging for the electrode array to achieve the intended depth. Furthermore, we observed significant fluctuations in the load when the implantation displacement was between 6 and 8 mm. This is due to the fact that the length of the electrode is only 10 mm, and towards the end of the implantation, the electrode is susceptible to the influence of minor vibrations from the loading platform and other factors, resulting in greater fluctuations in the force experienced by the electrode compared to the earlier stages. However, the overall trend of the load is upward.

**Figure 13:**
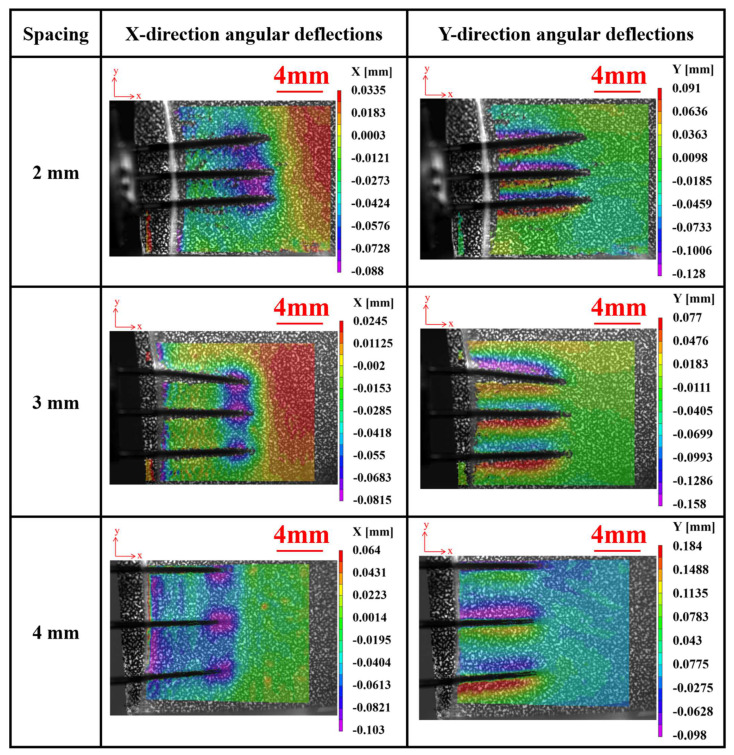
The X and Y direction angular deflections of agarose gel during array electrode implantation experiments with different spacings.

Figure 13 depicts the angular deflections of agarose gel in the X and Y directions during implantation experiments using electrodes with different array spacings at a velocity of 2.63 mm/s. It is observed that, similar to a single electrode, deformation primarily concentrates near the electrode tips. However, during the implantation process with array electrodes, tissues experience more pronounced indentations and exhibit an overall compressed state, contrasting with the implantation scenario of a single electrode. This phenomenon can be attributed to the coupling effect between adjacent electrodes, which gradually diminishes with increasing spacing. Further experimental observations indicate that when the spacing between electrodes increases to 4 mm, the coupling effect between them becomes weak and practically negligible.

During the implantation process, the effect of the electrode on the surrounding tissue is primarily limited to its immediate surrounding area. Increasing the spacing means the overlap between the areas of influence of each electrode decreases, thereby reducing the coupling between electrodes. This is very important for reducing tissue damage and improving the accuracy of implantation. Furthermore, as the spacing between the arrays increases, the overlap in the range of signals captured by each electrode decreases, reducing signal redundancy and mutual interference, thereby enhancing the accuracy and stability of long-term signal acquisition.

It can also be observed from Figure 13 that the array electrodes have exhibited an inclination to tilt inward or outward during the implantation process. Further observation reveals that this tilting of the electrodes predominantly occurs at the outermost electrodes. This is attributed to the fact that during the implantation of the array electrodes, as the depth of implantation increases, the brain tissue on both sides exerts a stronger boundary effect on the outermost electrodes. This leads to an imbalance in the forces acting on either side of the electrodes, resulting in the inward or outward tilting of the outermost electrodes. This suggests that during the implantation process of the array electrodes, the signals collected by the outermost electrodes may be affected by the boundary effect. This is particularly important for subsequent signal processing.

It should be emphasized that this study primarily focuses on providing a theoretical basis and experimental data support for the selection of electrode implantation speed and electrode array spacing during the process of electrode implantation into brain tissue. However, the research presented in this paper is limited to the application of the DGS system to transparent agarose gel models, which differs from the actual process of electrode implantation into non-transparent brain tissue. When applying the DGS system to observe the implantation of electrodes into non-transparent brain tissue, it is usually necessary to perform special surface treatments, such as coating, to enhance its observability within the DGS system [34,35]. Future research should fully consider the opacity of brain tissue and design new experiments based on the current experimental foundation. This includes selecting appropriate brain tissue models and further optimizing the DGS system, with the aim of achieving real-time observation of the electrode implantation process in biological brain tissue.

## 4. Conclusions

In conclusion, we prepared a highly transparent agarose gel with mechanical properties similar to those of biological brain tissue and developed an experimental setup based on the Digital Gradient Sensing (DGS) method to observe in real time the load and damage during the implantation of electrodes into the gel. The hyperelastic and viscoelastic properties of the gel were obtained through compression and indentation experiments, confirming its similarity to the mechanical properties of biological brain tissue. In the single electrode implantation experiments, as the implantation speed increased, both the degree of damage to the gel and the implantation load increased, while the area of damage near the electrode tip gradually decreased. However, if the implantation speed is too high, it may cause local damage that exceeds the gel’s tolerance threshold. The experiments suggest that an implantation speed of 2.63 mm/s may be appropriate as it strikes a good balance between the area of damage and the load. In the array electrode implantation experiments, as the spacing between electrodes increased, the implantation load rose, and the coupling effect between adjacent electrodes gradually diminished. When the electrode spacing was increased to 4 mm, the coupling effect became negligible, which is beneficial for enhancing the accuracy and reliability of long-term signal acquisition. These findings provide significant guidance for optimizing the parameters of array electrodes and the design of implantation experiments, and they lay a solid foundation for the clinical application of array electrode implantation into brain tissue.

## Data Availability

Data are contained within the article.

## Supplementary Materials

The following supporting information can be downloaded at: https://www.mdpi.com/article/10.3390/ma17102334/s1, Figure S1: Strain cloud diagrams at implantation speeds of (a) 1 mm/s, (b) 2.63 mm/s, (c) 5 mm/s. Figure S2: Load–displacement curves of experiments and simulations under different implantation speeds. Supplementary Videos S1 and S2.
