# Supplementary material for: Experimental Study of the Implantation Process for Array Electrodes into Highly Transparent Agarose Gel
Source: Materials (Basel). 2024 May 14;17(10):2334. doi: 10.3390/ma17102334 (PMC11123045; doi:10.3390/ma17102334)
Supplement: Supplementary file 1 [file materials-17-02334-s001.zip › materials-2978377-supplementary.pdf]

## Supplementary Material

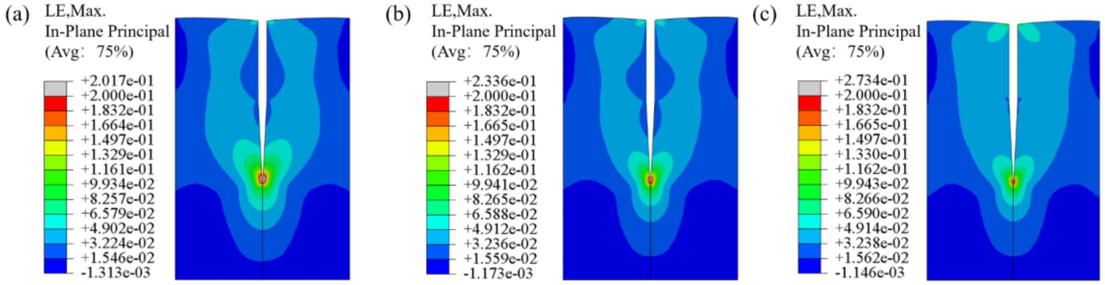


Figure. S1 Strain cloud diagrams at implantation speeds of (a)1 mm/s, (b)2.63 mm/s, (c)5 mm/s.

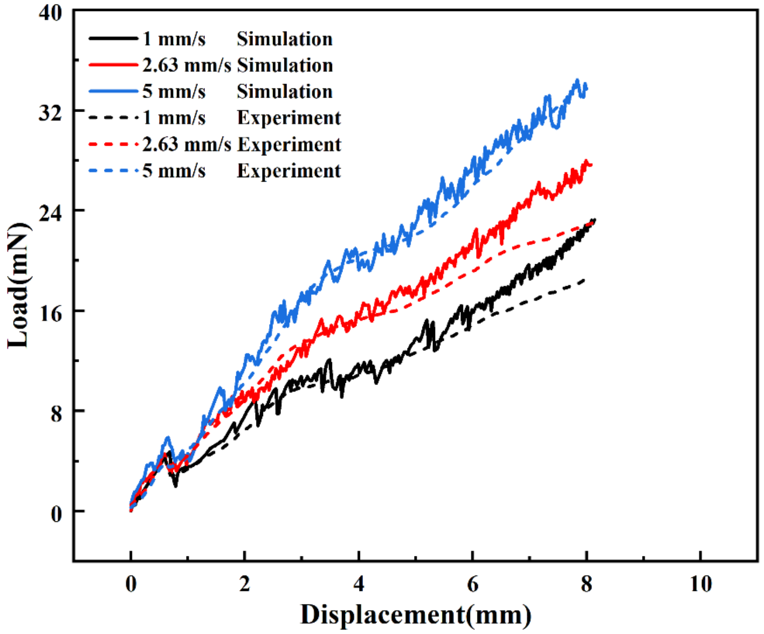


Figure. S2 Load-displacement curves of experiments and simulations under different implantation speeds.
